# Wearable Technology and Its Potential Role in Cardiovascular Health Monitoring and Disease Management

**DOI:** 10.1002/hsr2.71486

**Published:** 2025-11-19

**Authors:** Abubakar Nazir, Awais Nazir, Muhammad Shah Wali Jamal, Safi ur Rehman Sadiq, Shafaq Aman, Mubarak Jolayemi Mustapha, Sodiq Olatunbosun Lawal, Misbahudeen Olohuntoyin AbdulKareem, Mustapha Fatihi Bamigbola

**Affiliations:** ^1^ Department of Medicine King Edward Medical University Pakistan; ^2^ Department of Medicine The Jewish Hospital‐ Mercy Health, Cincinnati Ohio; ^3^ Oli Health Magazine Organization, Research and Education Kigali Rwanda; ^4^ Faculty of Basic Medical Sciences University of Ilorin Ilorin Nigeria; ^5^ Federal Medical Centre, Abeokuta; ^6^ Faculty of Clinical Sciences Obafemi Awolowo University Ile‐Ife Nigeria; ^7^ College Of Health Sciences University of Ilorin Nigeria

**Keywords:** artificial intelligence, cardiovascular disease, Internet of Medical Things (IoMT), precision cardiology, remote patient monitoring, telemedicine, wearable technology

## Abstract

**Background:**

Cardiovascular disease (CVD) remains the foremost cause of death globally, accounting for approximately 17.9 million deaths in 2019, 32% of all global fatalities with myocardial infarction and stroke constituting 85% of these. The disproportionate burden in low‐ and middle‐income countries underscores the urgent need for scalable, technology‐driven preventive and diagnostic strategies. Wearable technology represents a paradigm shift in cardiovascular health monitoring by facilitating continuous, real‐time physiological assessment and early disease detection.

**Objectives:**

To critically synthesize and appraise the current evidence on wearable technology for cardiovascular disease prevention, early detection, and long‐term management, emphasizing its clinical applications, integration with digital ecosystems, and implications for precision cardiology.

**Methods:**

A comprehensive, multi‐database literature search was conducted spanning January 2000 to 2025. The electronic databases PubMed, Scopus, Web of Science, ScienceDirect, IEEE Xplore, and Cochrane Library, along with Google Scholar for gray literature, were systematically queried. Search terms combined MeSH and free‐text keywords, including “wearable technology,” “cardiovascular monitoring,” “arrhythmia detection,” “heart failure,” “remote patient monitoring,” “artificial intelligence,” and “digital health.” Study selection followed PRISMA‐aligned screening principles, with inclusion criteria encompassing peer‐reviewed clinical trials, systematic reviews, and technology validation studies focusing on cardiovascular applications of wearable devices. Exclusion criteria included nonhuman studies, non‐English papers, and reports lacking clinical or technical relevance. Data extraction was independently performed by two reviewers, emphasizing device characteristics, monitoring parameters, diagnostic accuracy, patient outcomes, and integration with healthcare infrastructure. Methodological quality was appraised using standardized critical‐appraisal tools. Supplementary evidence was retrieved from the World Health Organization, U.S. FDA, and European Medicines Agency to contextualize regulatory and translational perspectives.

**Results:**

Wearable devices demonstrated robust capability in continuous cardiovascular monitoring, enabling early detection of arrhythmias, hypertension, heart failure, and ischemic events. Devices integrating photoplethysmography, ECG, biosensors, and machine‐learning algorithms significantly enhanced diagnostic sensitivity while improving patient engagement and lifestyle modification. Integration with telemedicine, artificial intelligence (AI), and the Internet of Medical Things (IoMT) enabled predictive analytics and personalized care. Major limitations included variability in data accuracy, interoperability barriers, regulatory heterogeneity, and privacy vulnerabilities. Nonetheless, aggregated evidence indicates substantial improvement in early detection, remote disease management, and patient outcomes.

**Conclusion:**

Wearable technology stands at the frontier of precision cardiovascular medicine, offering continuous, noninvasive, and data‐driven insights into patient health. The convergence of AI, IoMT, and real‐world data integration positions wearables as indispensable tools for risk stratification and remote management. Future work should prioritize standardized validation frameworks, cybersecurity reinforcement, and equitable global accessibility to maximize clinical impact and population‐level benefits.

## 1 Introduction

Cardiovascular diseases were responsible for 17.9 million deaths in 2019, representing 32% of all deaths worldwide. Heart attack and stroke deaths accounted for 85% of these fatalities and the majority of CVD fatalities occur in low‐ and middle‐income countries [1]. Wearable technology refers to the technology that is made to be worn which includes smartwatches and smart glasses. These electronic devices are adjacent to or on the surface of skin where they examine, ascertain and carry information and allow instant biofeedback to the wearer [2].

Wearable technology, particularly sleep trackers in the form of wristbands, headbands, sensor clips, in‐bed sensors, and other biofeedback devices, has become widely accessible without clinical authorization [3]. These devices can measure heart rate, skin conductance, temperature, and behavioral data such as sleep patterns [3, 4, 5]. Their advantages include continuous, passive data collection without active user input or manual data processing. Moreover, by providing personalized feedback, they hold potential to enhance patient engagement, promote healthier routines, and support early detection of health changes. However, questions remain about the accuracy and reliability of the data they generate, especially when used for medical decision‐making [2, 3, 4].

These technologies can be used for making clinical diagnosis by gathering the data over a long duration and can bring down the expenses on hospitalization. Cardiologists in the US are using this technology to give diagnosis via these wearable devices and give health based solutions [3]. Early identification of the critical medical events allows patients more time to look for medical consultation. Remote monitoring can further help in development of the implantable cardioverter‐ defibrillator and decreased incidences of irregular shock related to these devices [3, 4].

Wearable technologies have increasingly incorporated advanced sensors capable of continuous or intermittent noninvasive blood pressure monitoring, representing a significant innovation in cardiovascular risk management. Techniques such as pulse transit time (PTT) and photoplethysmography (PPG) enable estimation of blood pressure metrics without the need for traditional cuff‐based devices, facilitating ambulatory and real‐world data acquisition. This continuous monitoring capacity allows for improved detection of hypertensive episodes and blood pressure variability, which are critical in prognosticating cardiovascular events [2, 6, 7, 8].

Vascular age is widely recognized as a superior predictor of cardiovascular morbidity and mortality compared to chronological age alone [5, 8]. While direct measurement of vascular age typically necessitates specialized vascular imaging or tonometry‐based assessment of pulse wave velocity (PWV), emerging wearable platforms seek to estimate vascular age indirectly by leveraging surrogate hemodynamic parameters derived from sensor data, including PWV estimates and blood pressure trends. The integration of vascular age assessment into wearable devices holds promise for personalized cardiovascular risk stratification and early intervention [8].

Significant improvement and development have been made in processing of these devices which increases the accuracy and greater authenticity of the information helping to diagnose a particular disorder or to differentiate between multiple disorders.

### 1.1 Wearable Technology in Prevention and Lifestyle Modification

**Figure 1:**
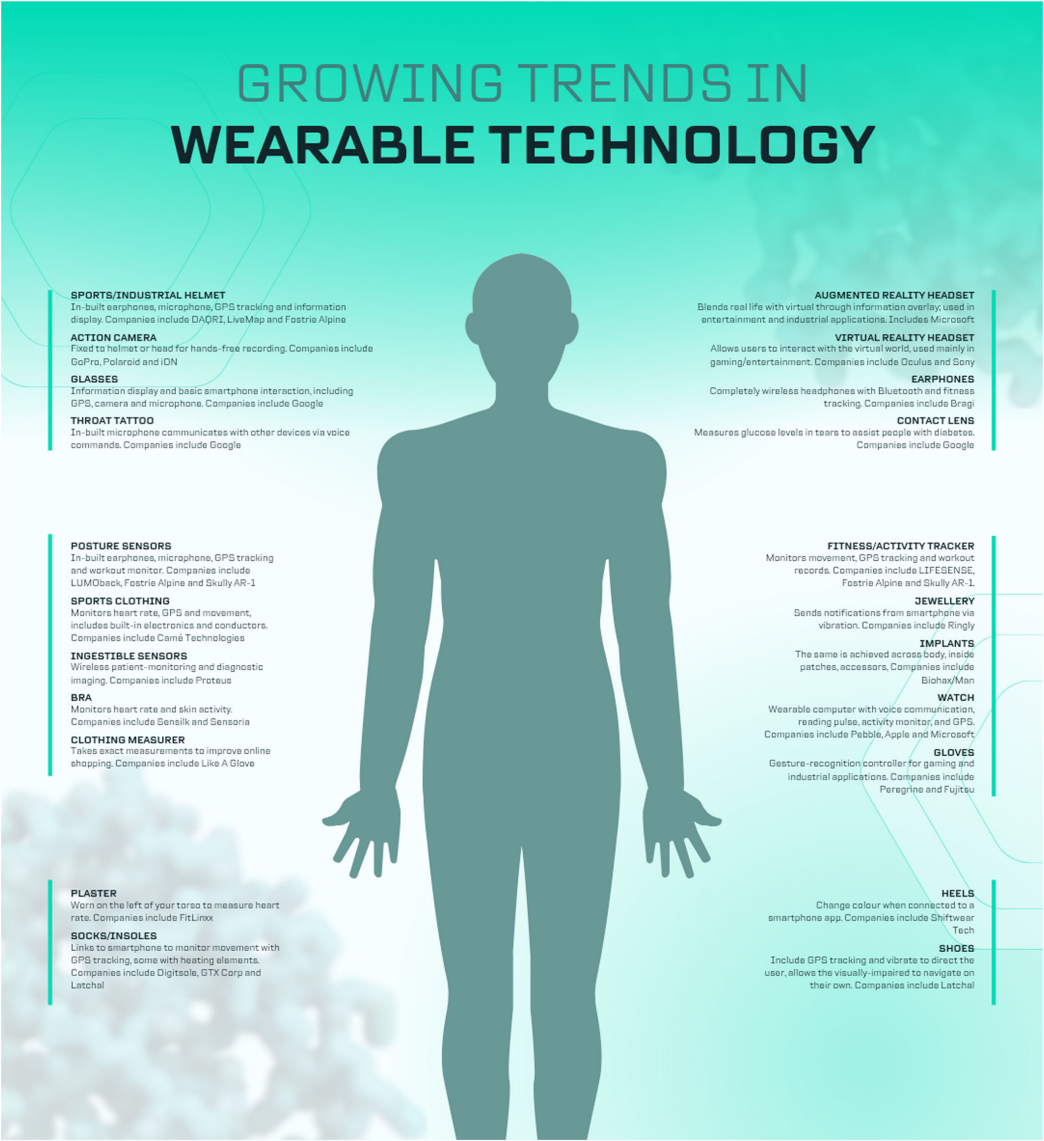
Wearable technology in prevention and lifestyle modification.

Changing behavior related to improper lifestyle habits has attracted attention as a solution to prevent lifestyle diseases, such as diabetes, heart disease, arteriosclerosis, and stroke [5]. To drive health behavior changes, wearable devices are needed, and they must not only provide accurate sensing and visualization functions but also effective intervention functions (Figure 1).

Goal setting has shown some promise in promoting dietary and physical activity behavior change among adults, but methodological issues still need to be resolved. The literature with adolescents and children is limited, and the authors are not aware of any published studies with this audience investigating the independent effect of goal setting on dietary or physical activity behavior [6]. Although goal setting is widely used with children and adolescents in nutrition interventions, its effectiveness has yet to be reported.

Wearable and mobile sensor technologies can be useful tools in precision nutrition research and practice, but few are reliable for obtaining accurate and precise measurements of diet and nutrition. A study documents high variability in the accuracy and utility of a wristband sensor to track nutritional intake, highlighting the need for reliable, effective measurement tools to facilitate accurate, precision‐based technologies for personal dietary guidance and intervention [7, 8].

Smartwatches equipped with heart rate variability (HRV) monitoring capabilities play a valuable role in various activities and stress management. Wearables like Apple Watch, Garmin Forerunner, WHOOP Strap, Fitbit Sense, and Oura Ring use HRV monitoring to assess autonomic balance, guide stress management, and optimize physical activity [9, 10, 11, 12]. These devices provide real‐time feedback on recovery and stress, enabling personalized behavior modification. Their validated HRV measurements support both athletic performance and general wellness interventions [9, 10, 11, 12]. These devices offer a range of features, including activity tracking, heart rate monitoring, sleep tracking, and stress management interventions based on HRV data. Its HRV data has provided a very useful information of personal health regarding level of stress, self‐management of stress factors and understanding of sleep and personal health. By providing real‐time feedback, promoting self‐awareness, and offering personalized stress management techniques, smartwatches empower individuals to monitor and manage their stress levels effectively. Integrating smartwatch HRV data with professional medical advice and personalized care can ensure a comprehensive and effective approach to stress management [8].

Efficient behavior change in non‐sport patients through wearable technology relies on integrating continuous self‐monitoring with personalized, theory‐driven feedback to enhance motivation and self‐efficacy [3, 6]. Utilizing behavioral frameworks such as the Social Cognitive Theory, effective interventions incorporate goal‐setting, real‐time prompts, and social support to promote gradual increases in physical activity [3, 7, 8]. Combining wearables with tailored coaching or digital health interventions enhances engagement and adherence by addressing individual barriers and reinforcing positive habits (Stephens et al. 2018). Ultimately, sustained behavior change requires adaptive, multi‐component strategies that dynamically adjust feedback and support according to user progress and preferences [5, 6, 7, 8].

### 1.2 Wearable Technology in Cardiovascular Health Monitoring

**Figure 2:**
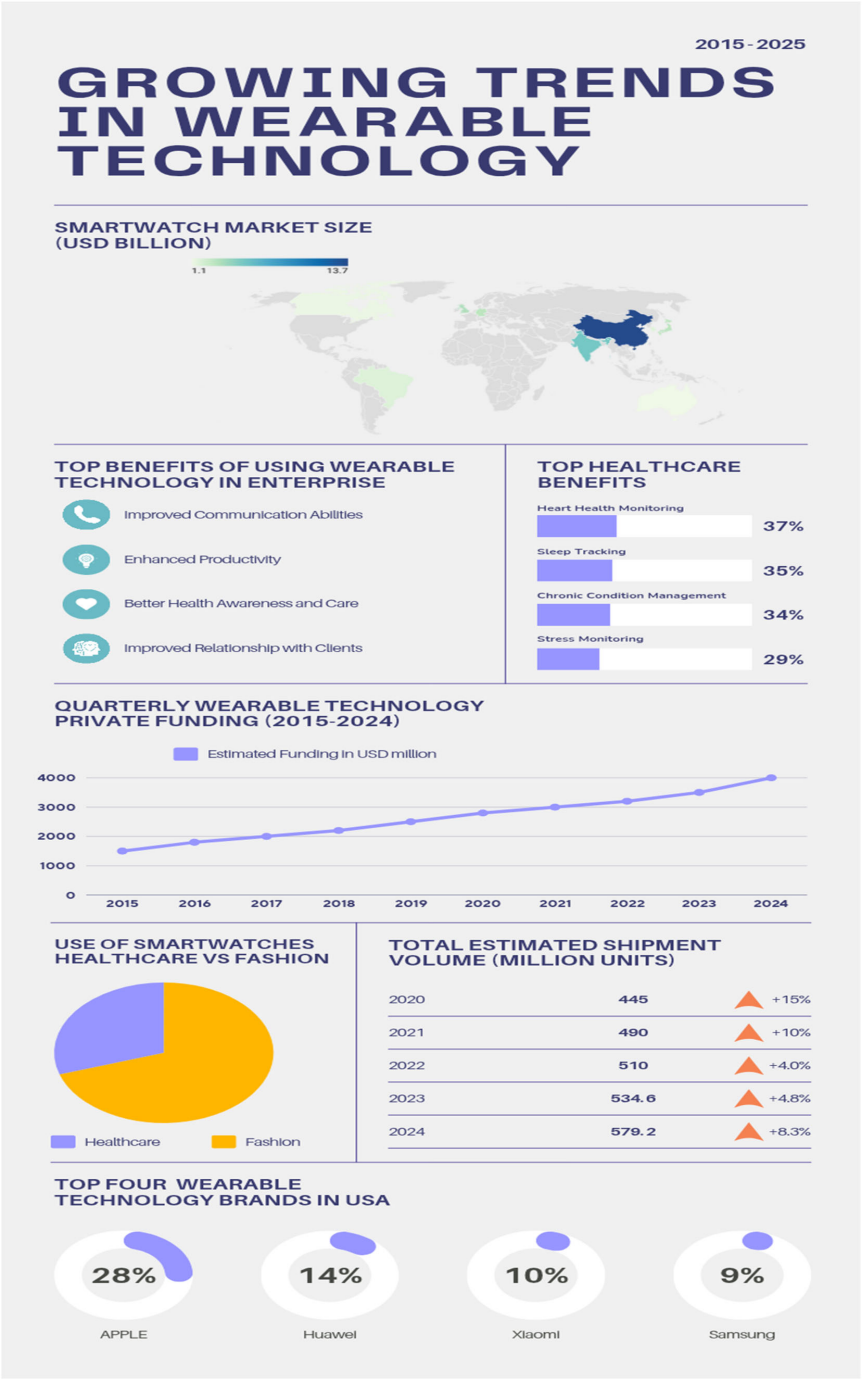
Wearable technology in cardiovascular health monitoring [2,5,8].

Commercial wearables usually include Smartwatches and fitness trackers, Heart rate monitors and chest straps, ECG/EKG devices, Smart clothing and patches smart wristbands, patches, and they generally monitor variables such as heart rate, blood oxygen saturation, and electrocardiogram data. Noncommercial wearables focus on monitoring electrocardiogram and photo plethysmography data, and they mostly include accelerometers and smartwatches for detecting atrial fibrillation and heart failure (Figure 2).

**Figure 3:**
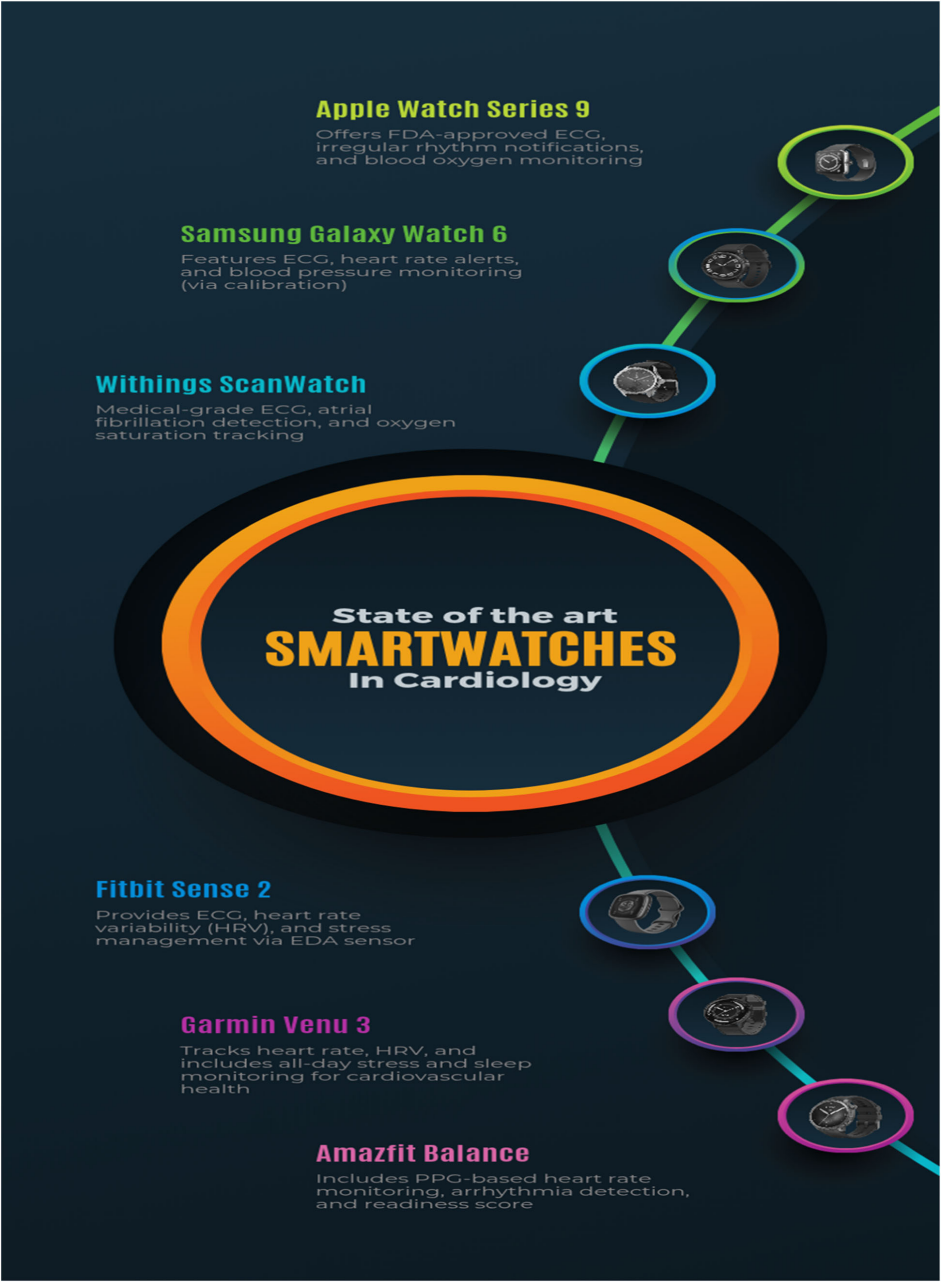
Types of smartwatches in cardiology.

Features and capabilities of wearable devices include among others, continuous heart rate monitoring, Blood pressure monitoring, electrocardiogram (ECG/EKG) recording, sleep tracking and analysis, physical activity and exercise monitoring. VITAL‐ECG (vitalsignscorp.com) is a smartwatch, developed to perform the most used checks as a “one touch” device, anywhere, at low cost. “One touch” because the postoperative person should not be an expert in medical devices and our medical smartwatch can be used with just one finger [8] everywhere because he can live away from the hospital. It has a relatively low cost because anyone can afford to use it. It is a wearable and easy‐to‐use device that works with any tablet or smartphone to monitor the most important vital parameters: electrocardiogram and heart rate, blood oxygen level, skin temperature and moisture, and physical activity of the patient. Machine learning algorithms can detect anomalies in the patient's state and report it to medical staff via the smartphone. Among other capacities and features of other wearable devices, this just shows how efficient and useful they can be (Figure 3).

### 1.3 Wearable Technology in Cardiovascular Disease Management

**Figure 4:**
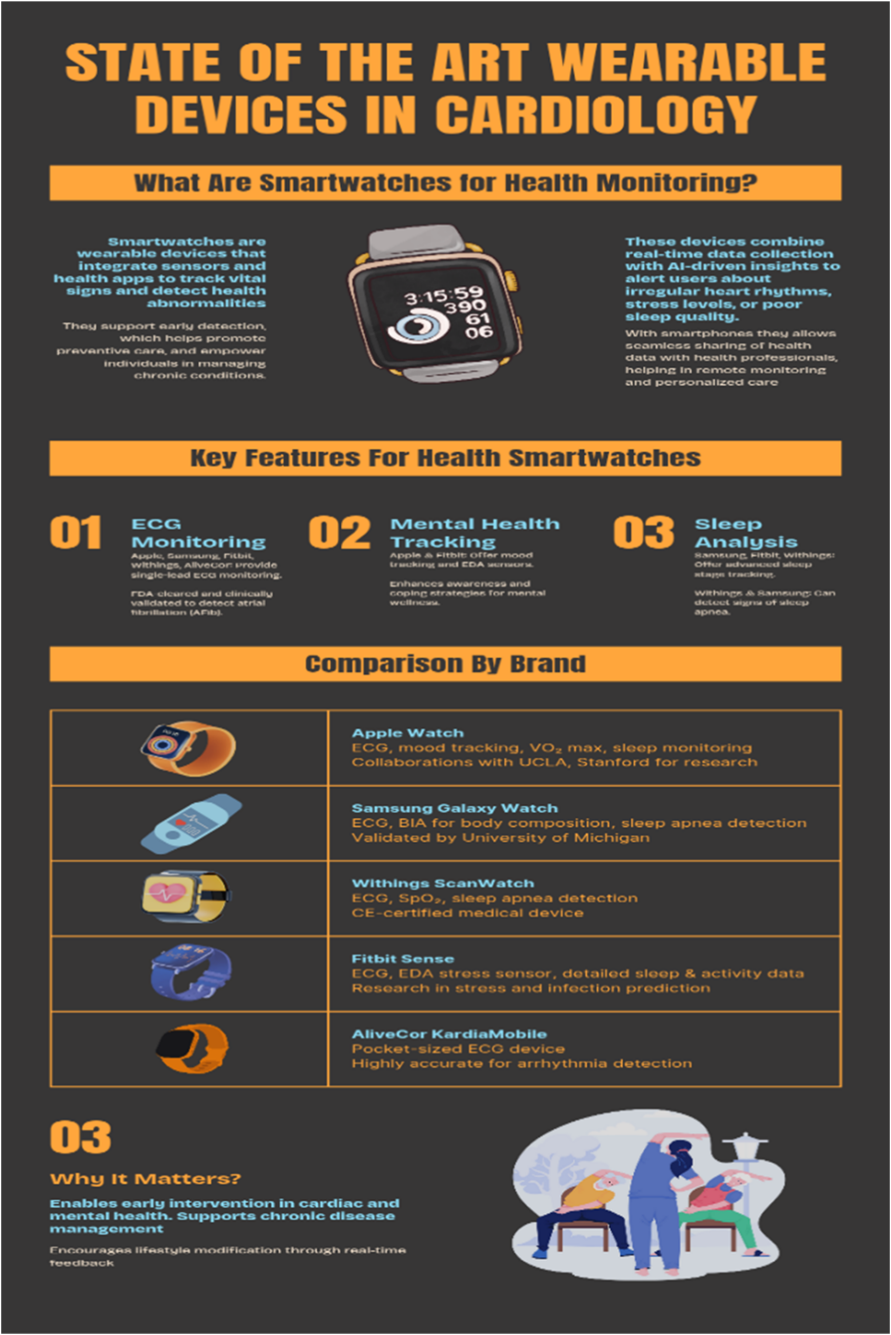
State of the art wearable devices in cardiology.

Cardiovascular diseases require continuous monitoring of vital signs and cardiovascular status to reduce morbidity and mortality by enabling timely diagnosis, preventing complications, and guiding management [9]. These diseases often represent a spectrum, beginning with primary hypertension and potentially progressing to heart failure, arrhythmias, and cerebrovascular conditions. Continuous, up‐to‐date recording of cardiovascular parameters is essential to promptly detect complications and monitor patients' well‐being. Diagnosis of hypertension—a lifelong condition—requires adherence to specific guidelines, particularly in patients with borderline blood pressure, often utilizing ambulatory blood pressure monitoring (ABPM). The advent of wearable technologies has facilitated this process by enabling 24‐h blood pressure measurements without inconveniencing patients, thus improving monitoring and complication prevention; for example, elevated morning blood pressure is linked to increased stroke risk, whereas elevated nighttime pressure correlates with target organ damage [10]. Among blood pressure monitoring methods, oscillometric devices remain the most widely used due to their validated accuracy, reproducibility, and ease of use in ambulatory and home settings. However, emerging technologies such as ultrasound‐based devices and PPG offer promising cuffless, continuous, and noninvasive monitoring options. Ultrasound provides direct arterial wall assessment and hemodynamic data, making it particularly useful for vascular age estimation and central blood pressure measurement, which are superior predictors of cardiovascular risk compared to peripheral measurements. PPG, incorporated into many smartwatches (e.g., Apple Watch, Garmin, Fitbit), utilizes optical sensors to detect volumetric changes in blood flow, facilitating heart rate and arrhythmia detection with growing accuracy, though its performance can be affected by factors such as skin tone and ambient light interference [13, 14] (Table 1) (Figure 4).

**Table 1:** Advancements in wearable technology for cardiovascular health monitoring and disease management.

Device type	Technology integrated	Cardiovascular parameters monitored	Clinical applications	Advantages	Limitations
Smartwatches (e.g., Apple Watch, Fitbit, Garmin, Samsung Galaxy Watch)	Photoplethysmography (PPG), single‐lead electrocardiography (ECG), accelerometers, AI‐based health algorithms	Heart rate (HR), heart rate variability (HRV), blood oxygen saturation (SpO₂), activity levels, atrial fibrillation (AFib) detection	Early arrhythmia detection, AFib monitoring, stress analysis, general fitness tracking	Noninvasive, real‐time continuous monitoring, widely accessible, user‐friendly interfaces, and integration with mobile health applications	Limited ECG accuracy compared to multi‐lead systems, motion artifacts, dependency on user compliance, potential for false positives
Continuous ECG Patches (e.g., Zio Patch, Cardea SOLO, Holter monitors)	Multi‐lead ECG electrodes, AI‐driven arrhythmia detection	Multi‐lead ECG, HRV, arrhythmia (AFib, ventricular tachycardia, bradycardia)	Long‐term arrhythmia monitoring, post‐myocardial infarction (MI) follow‐up, stroke risk assessment	Higher accuracy than smartwatch‐based ECG, prolonged continuous monitoring (up to 14 days), high compliance due to unobtrusive design	Single‐use (in some cases), cost‐intensive, potential for skin irritation, data analysis requires clinical review
Wearable Blood Pressure Monitors (e.g., Omron HeartGuide, Aktiia, Biobeat)	Oscillometric method, tonometry‐based blood pressure measurement, AI‐based trend prediction	Systolic and diastolic blood pressure, HR	Hypertension management, cardiovascular risk assessment, stroke prevention	Clinically validated, portable, real‐time BP tracking, provides long‐term BP trends	Bulky design, intermittent measurements, requires calibration, affected by movement artifacts
Smart Rings (e.g., Oura Ring, Circular Ring, Motiv Ring)	PPG sensors, temperature monitoring, HRV analysis, sleep cycle tracking	HRV, SpO₂, resting HR, sleep patterns	Early cardiovascular risk stratification, autonomic nervous system monitoring, recovery assessment	Small, discreet, longer battery life than smartwatches, continuous physiological tracking	Limited cardiac‐specific features, small sensor area leading to potential inaccuracies
Wearable Biosensors (e.g., VitalPatch, BioPatch, MC10 BioStamp)	Flexible biosensors, AI‐driven predictive modeling, real‐time telemetry	ECG, respiration rate, temperature, motion analysis, HRV	Continuous ICU‐level cardiac monitoring, remote patient management, personalized medicine	High‐fidelity physiological data capture, wireless and Noninvasive, allows early intervention	Short battery life, potential for skin irritation, requires integration with clinical workflows
Smart Clothing (e.g., Hexoskin, Myant, Siren Socks)	Textile‐integrated ECG electrodes, PPG sensors, respiratory rate sensors, impedance cardiography	Multi‐lead ECG, respiration rate, HR, HRV, temperature, movement analysis	Post‐MI rehabilitation, remote patient monitoring, stress analysis	Comfortable for long‐term use, multi‐parameter data collection, high compliance	Expensive, requires frequent washing/maintenance, integration challenges with clinical systems
Implantable Loop Recorders (e.g., Reveal LINQ, BioMonitor 2, Confirm RX by Abbott)	Subcutaneous ECG sensors, continuous wireless telemetry, cloud‐based AI‐driven arrhythmia detection	Continuous ECG, long‐term arrhythmia detection (AFib, bradycardia, tachycardia), ischemic event monitoring	Cryptogenic stroke diagnosis, long‐term cardiac event detection, syncope assessment	Highly accurate, long battery life (~3 years), does not require patient activation	Invasive, high cost, requires implantation by a specialist, may not detect transient non‐arrhythmic abnormalities
Wearable Sweat & Biochemical Sensors (Emerging Technologies, e.g., Gatorade GX Sweat Patch, Graphene‐based electrochemical sensors)	Electrochemical biosensors, sweat analysis, noninvasive biomarkers	Electrolytes, lactate, glucose, cortisol, pH levels	Noninvasive metabolic and cardiovascular stress assessment, dehydration risk detection	Provides biochemical markers without blood sampling, real‐time data collection, potential integration with AI‐driven predictive analytics	Limited cardiovascular specificity, affected by hydration levels and sweat composition variability

An arrhythmia is a deviation from the sinus rhythm, traditionally diagnosed using an electrocardiogram. In patients with increased tendencies to develop arrhythmia, wearable technologies have been used to detect any rate abnormality as soon as possible. In patients diagnosed, some of these technologies have been used to monitor drug response and lower the risk of complications. Digital devices have been used extensively in the assessment of atrial fibrillation, which is one of the most common arrhythmias seen in clinical practice, using the ABC‐integrated strategy, which involves – Avoidance of stroke, Better symptoms management and Cardiovascular and other comorbidities risk reduction, making wearables technology gradually indispensable in the management of arrhythmias [10] (Table 1).

The use of wearable devices in managing cardiovascular diseases, particularly heart failure, is pivotal for slowing disease progression, preventing complications, and detecting early signs of heart failure [11]. These devices enable remote monitoring through continuous data collection and analysis outside traditional clinical settings, allowing for timely adjustments in medication based on real‐time readings. However, integrating and securing the data generated by these devices poses challenges related to data privacy, accessibility, and interoperability across different healthcare units [12]. To address these issues, technologies like artificial intelligence (AI), machine learning (ML), and block chain are employed for secure data interpretation and management. While electronic health records (EHRs) already incorporate patient data, integrating information from health wearable technology (HWT) promises to improve healthcare delivery and support medical research. Nonetheless, a feasibility study highlighted shortcomings in clinician‐patient communication and feedback loops using HWT, revealing that physicians often fail to provide necessary follow‐up based on device data, which can impact patient satisfaction negatively [15, 16] (Table 2).

**Table 2:** Comparison of traditional health monitoring devices and wearable devices for cardiovascular health.

Feature	Traditional health monitoring devices	Wearable devices
Mode of use	Used in clinical settings or at home with manual operation	Continuous, real‐time monitoring during daily activities
User accessibility	Requires professional assistance or periodic self‐use	User‐friendly, designed for independent use
Monitoring capabilities	Intermittent measurements (e.g., blood pressure cuffs, ECG machines)	Continuous tracking of heart rate, ECG, oxygen levels, activity, etc.
Data collection	Manual recording or stored in device memory	Automatic storage, cloud‐based access, and integration with mobile apps
Early detection of abnormalities	Detected only during scheduled check‐ups	Real‐time alerts for arrhythmias, hypertension, and other anomalies
Portability	Limited portability (e.g., bulky monitors, wired ECG)	Compact, lightweight, and wearable (e.g., smartwatches, patches)
Patient compliance	Dependent on patient adherence to routine check‐ups	Higher compliance due to ease of use and real‐time feedback
Data transmission	Requires manual reporting or hospital visits	Wireless data transmission to healthcare providers via apps
Intervention timing	Delayed response due to infrequent monitoring	Immediate response with alerts for critical conditions
Examples	Holter monitors, sphygmomanometers, ECG machines	Smartwatches (Apple Watch, Fitbit), biosensors, chest patches

For arrhythmia detection and monitoring, wearable ECG devices like the AliveCor KardiaMobile, Fitbit Sense, and Apple Watch Series (with FDA clearance for AFib detection) have revolutionized early identification and ongoing management of atrial fibrillation and other rhythm disorders. These technologies support the ABC strategy (avoid stroke, Better symptom control, cardiovascular risk reduction) by enabling timely intervention and monitoring therapeutic response [10]. Continuous heart failure monitoring devices such as the CardioMEMS HF System represent an advanced approach by employing implantable sensors to track pulmonary artery pressures remotely, allowing proactive adjustments in therapy to prevent hospitalizations and disease progression [11].

Despite these technological advances, significant challenges remain regarding the integration of wearable‐generated data into clinical workflows, ensuring patient data privacy, and achieving interoperability across disparate healthcare systems and EHRs [12]. AI and machine learning algorithms are increasingly employed to process and interpret large volumes of longitudinal data from wearables, enhancing predictive accuracy and enabling personalized clinical decision‐making. However, studies reveal persistent gaps in clinician‐patient communication and insufficient feedback loops based on wearable data, which may hinder patient engagement and satisfaction, emphasizing the critical need for streamlined data sharing protocols and clinician education to maximize clinical utility [15, 16].

In summary, while oscillometric devices currently provide robust and widely accepted blood pressure monitoring for hypertension diagnosis and management, ultrasound and PPG technologies offer promising avenues for continuous, noninvasive cardiovascular assessment with greater physiological insight. Device selection should be guided by the clinical context: oscillometric cuffs remain preferred for formal hypertension diagnosis; PPG‐enabled wearables are ideal for arrhythmia screening and general wellness monitoring; and implantable or ultrasound‐based devices are best suited for advanced heart failure management and vascular assessments requiring detailed hemodynamic data.

### 1.4 Integration of Wearable Technology With Other Healthcare Technologies

Internet of Things and Connected Healthcare Devices or Internet of Medical Things (IoMT), as they are recently called, is the blend of medical devices with the Internet of Things (IoT). IoMTs are used to refer to “things” within which are sensors or software used to exchange data with other devices across the internet and are monitored by healthcare professionals. These devices have been largely employed in healthcare delivery, significantly changing the game's dynamics. Some of the common ways in which IoMTs have been used include Remote Patient Monitoring, Heart rate monitoring, Glucose monitoring, hand‐hygiene monitoring, depression and mood monitoring, controlled inhalers, Parkinson's disease monitoring, and even robotics surgery. It as well found its use during the COVID‐19 pandemic due to the lockdown [13]. (Table 2)

Here is a comparative table between traditional health monitoring devices and wearable devices, highlighting their key differences in cardiovascular health monitoring and disease management:

Mobile health (mHealth), the widely embraced form of IoMTs with the use of mobile phones, are found in virtually all aspects of health, ranging from appointment reminder to treatment compliance, health call centers, patient monitoring, surveillance, and fitness, among others [14]. Some common mHealth applications and platforms include; HealthTap, WebMD, Generis, Pocket Pharmacy, Teladoc, Mayoclinic, etc. [17].

mHealth is only one of the most common technology used among others, generally referred to as Telemedicine, which uses technology to diagnose, treat and monitor patients. Telemedicine stems from a bigger term, telehealth which deals with a much wider exposition of healthcare delivery and services. Telemedicine was birthed from the need for remote consultation and follow‐up, and it has gained a lot of ground and embracement by doctors and patients alike. An important aspect of Telemedicine, Remote Patient Monitoring (RPM) deals with getting up‐to‐date patient‐generated data and delivering it to their healthcare professional making healthcare delivery and prevention of complications much easier. RPM technology ranges from handheld devices to mobile apps and online platforms; the common one includes blood pressure and health rate monitoring, blood glucose monitoring, calorie intake and diet logging, sleep logging etc. [18].

Telemedicine, in general, seeks to reduce patient waiting time, prevent long hospital admission, increase access to specialists, prompt response in emergencies, prevent complications and manage and monitor patients with chronic diseases, among others. These have been largely accomplished, with the main concern being patient data security.

Alongside data security, interoperability of the different telehealth appliances with patient records has been a major topic of debate, and much research has gone into this. There have been many technologies, the most frequently used being the wearables that take patients' vitals passively. Many health institutions have started incorporating these data into patient portals, making follow‐up easy. The wearable technology also provides a platform from which the patient's data can be assessed against the traditional use of desktops [19].

Patient data security within telemedicine, particularly concerning wearable devices, is maintained through a combination of advanced encryption methods, standardized data exchange protocols, and stringent regulatory compliance [20]. Wearables collect sensitive health information that must be securely transmitted and stored to prevent unauthorized access and breaches. Encryption standards such as Advanced Encryption Standard (AES) protect data at rest, while Transport Layer Security (TLS) secures data in transit between devices, patient portals, and healthcare systems. The Health Level Seven International Fast Healthcare Interoperability Resources (HL7 FHIR) protocol ensures secure, standardized interoperability between wearables and EHRs, facilitating seamless yet protected data exchange [20]. Authentication mechanisms, including multi‐factor authentication (MFA) and OAuth 2.0, control user access, verifying identities before permitting data retrieval or entry. Compliance with legal frameworks such as HIPAA and GDPR mandates rigorous safeguards for patient privacy and data security, ensuring that telemedicine platforms and wearable technologies uphold the highest standards for protecting personal health information [20]. These combined measures create a secure environment that preserves patient confidentiality while enabling real‐time monitoring and remote healthcare delivery.

### 1.5 Case Studies and Success Stories

Numerous studies have documented the impact of wearable and telemedicine in healthcare delivery, especially cardiovascular care. In 2019, a study assessed using a smartwatch to identify atrial fibrillation. More than 400,000 patients were recruited for the study, and it was concluded afterward that 84% of irregular pulse notifications were concordant with atrial fibrillation [6]. Another study, a systematic review, shows the importance of wearable devices in monitoring patients with heart failure and found a significant increase in quality of life, reduced heart failure‐related mortality and reduced hospital admission in patients remotely monitored using wearable devices than other patients [20].

It was also found in a few other studies that remote monitoring has significantly reduced in‐hospital patient admission and consequently increased the efficiency of healthcare delivery, especially in managing chronic diseases such as health failure. Patients with Heart Failure with reduced Ejection Fraction (HFrEF) can now have implantable cardiac devices which can be wirelessly connected to home monitors through which relevant alerts can be received [21]. They have also been proven to have high efficacy and significantly improved patient outcomes, contributing to increased use of these devices [22].

Various factors targeted by this technology and their relevance in the diagnosis and management of cardiovascular diseases, such as physical activities, sleep, and vital signs, have largely been analyzed and concluded to be effective and accurate as some of the factors yet to be harnessed, such as behavior change strategies among others have been descended and there's increasing expectation as technology capabilities increase [23].

As much as most of these technologies have generally improved patients' outcomes and healthcare delivery, they are largely customer‐driven, making it a flourishing investment for new producers to venture; thereby, the risk of counterfeiting is valid. There is a need to have an adequate regulatory oversight policy to ensure safety and efficacy through comprehensive evaluation frameworks of these products [24]. A 100% efficacy is understandably unlikely due to the difference in the makeup of different individuals and disease characteristics. Novel technologies have the potential to revolutionize every level of disease prevention by effecting meaningful and sustainable behavioral change in individuals [25].

Wearable technology is here to stay as it gets steadily sophisticated to cater to rising needs with a significant need for innovations in infant safety and care, elderly care, military support and preventive medicine. Data security and privacy, system operation cost, regulatory requirements and subpar adoption rates remain the major concerns.

### 1.6 Potential Impact of Wearable Technology

Smart wearable devices for health monitoring are highly applicable in cardiovascular health and medicine as a whole. The evolution of this technology has led to the development and optimization of wearable health‐monitoring systems and advancement of composite materials and system integration. The fabrication of miniaturized, noninvasive devices including smart watches, glasses and head bands with high grade sensor technology are now on the rise (including the formation of textile based and Tattoo Based HWDs based on the Epidermal electronic sensors or the Piezoelectric sensors for real‐time estimation of the Arterial Pulses, Rhythm, rates etc. in real time [26].

Piezoelectric sensors, which generate electrical signals in response to mechanical stress, are increasingly utilized in wearable cardiovascular monitoring devices, particularly those employing ultrasound technology for blood pressure assessment [26]. In wearable ultrasound systems, piezoelectric transducers emit and receive high‐frequency sound waves that interact with arterial walls, enabling continuous, noninvasive measurement of arterial diameter changes and PWV, key parameters for estimating blood pressure and vascular health [26]. This approach offers advantages over traditional cuff‐based oscillometric methods by allowing cuffless, real‐time monitoring that can be integrated into flexible, wearable formats such as armbands or patches. Furthermore, the high sensitivity and rapid response of piezoelectric sensors facilitate accurate detection of subtle hemodynamic variations, enhancing early detection of hypertension and cardiovascular risk stratification [26].

Novel wearable devices that quantify remote dielectric sensing (ReDS) and bioimpedance may identify preclinical changes in intravascular volume status. This could enable early intervention in decompensated Cardiac failure. Similarly, another HWD, Oura Ring a metallic ring that has miniaturized sensors to monitor physiological parameters, such as heart rate, body temperature, and respiratory rate. It is useful in heart failure as well as in Patients with SARS‐CoV and Community acquired pneumonia [27]. AI can be used to analyse data from sensors of wearable devices to provide an earlier and more accurate prediction and diagnosis and monitoringof cardiovascular diseases. This may help in disease prevention and thus the reduction of morbidity and mortality world wide. AI is a potential solution for precision medicine that is to diagnose and manage patients in a manner that is tailored towards their specific needs. More efforts will however be made to make this closer to reality by combining EHR, patient sensor data, and genomics using machine learning analytics. Detection and prediction of Cardiovascular diseases can be achieved by referencing data based on prior physiological Knowledge. Combination of data by AI can help professionals make more informed decisions as to the best care plan for individualized patients as behavioral, social and other exogenous determinants can now be tracked by wearable sensor based devices in real time which will provide data that will vary with every individual patient and can greatly influence decision making by Healthcare professionals. This can also assist patients in making informed decisions about their lifestyle choices aiding Healthcare and improving prognosis of diseases [28].

Finally, wearable technology in addition to providing and accumulating data will help in monitoring the health status of the population which will greatly help in the prevention of cardiovascular diseases. Sensors such as Accelerometers, Electrocardiogram, Heart rate monitors will note changes in human physiological activities including the level of physical activity, sleep pattern and HRV, furnishing continuous surveillance of vital signs thereby facilitating early detection of risk factors associated with Cardiovascular diseases and encouraging proactive management measures.

### 1.7 Challenges and Consideration in Wearable Technology

Significant draw backs however, to the progress and evolution of wearable technology should be taken into consideration.

A key concern is Data Quality, data to be evaluated must be of utmost quality to be able to back up the required scientific deductions. Data quality is one of the fundamental values of research ethics. High quality data results in positive clinical results. An issue that makes it so difficult to assess the quality of data when it comes to wearable devices is the degree of variability involved. Different sensors and different kinds of devices collecting data under different physical and environmental conditions will pose a significant challenge in Quality control [27, 29]. Also, interoperability standards are also very crucial for an effective and seamless use of data from wearable technology. This is currently very challenging as there is additional cost of integrating this data with the Healthcare systems including the employment of skilled staff to oversee the logistic demands including training patients on the use of wearables, analyzing data from different sources, managing the issue of cyber risks [27, 28]. Privacy and Data security concerns arise when issues concerning research on wearable technology are conducted as wearables collect personalized and sensitive data. There is need for infrastructure in research therefore, that will appropriately anonymize and encrypt patient information to conform to the required ethics. For example, a major security concern with a device like google fitbit is the lack of Authentication on the tracker leaving the wearer at the mercy of having their data being potentially stolen [29]. Wearable technology are prone to cyber‐attacks which pose a great risk to the patient. Cardiac implants can simply be turned off using electromagnetic interference and cause death. Also, a stolen Wearable device can pose significant risk of personal data leakage to the owner of the device [29, 30, 31, 32, 33, 34, 35].

**Figure 5:**
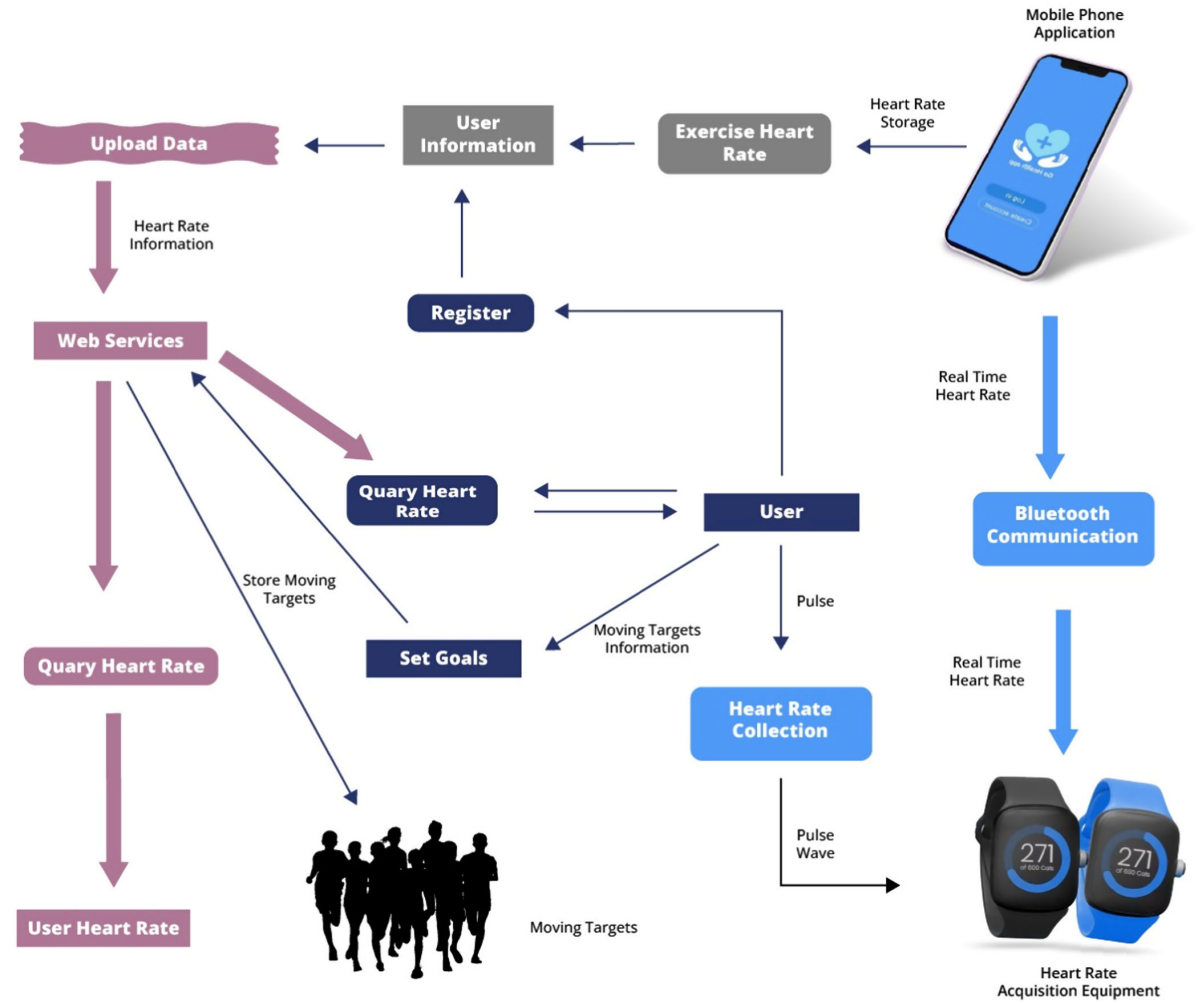
Utilizing digitally driven technologies in field of cardiovascular medicine.

Wearable devices face several limitations in their application for cardiovascular monitoring, primarily due to dependency on reliable power sources [35, 36, 37, 38, 39, 40, 41, 42, 43, 44, 45]. Power outages can lead to data loss and inaccurate readings, hindering their effectiveness. User engagement and competence present another challenge, especially among elderly populations who may struggle with technology usage, privacy concerns, and lifestyle compatibility. The power source is a critical component in the design and commercialization of wearable medical devices, significantly impacting device size, usability, and regulatory compliance [46]. Developers must balance energy capacity, device longevity, weight, and safety to ensure continuous, reliable operation without frequent recharging or replacement, which could hinder user adherence. Miniaturization of power sources is achieved through advancements in battery chemistry such as lithium‐polymer and solid‐state batteries that offer higher energy densities in smaller form factors [46]. Additionally, energy‐harvesting technologies, including piezoelectric, thermoelectric, and photovoltaic systems, are being explored to supplement or replace conventional batteries, enabling extended device autonomy and reducing dependence on bulky power supplies [46]. From a regulatory perspective, agencies like the U.S. Food and Drug Administration (FDA) and the European Medicines Agency (EMA) require comprehensive evaluation of power source safety, electromagnetic compatibility, and reliability under intended use conditions. Standards such as IEC 60601‐1 for medical electrical equipment specify criteria for battery safety, thermal management, and protection against electrical hazards [46]. Consequently, the choice and design of the power source must align with both technical performance and regulatory mandates to ensure patient safety, device efficacy, and market approval. Ethical considerations surrounding data transmission, device ownership versus data ownership, and the need for informed consent further complicate widespread adoption and research use of wearables [47, 48, 49, 50, 51, 52]. Cost and affordability also pose significant barriers, particularly in developing countries where resources for sensor‐based devices and data logistics are scarce [53, 54, 55, 56, 57, 58, 59]. Despite these challenges, the potential for wearable technology to revolutionize personalized healthcare and improve cardiovascular disease management globally remains promising, emphasizing its role in advancing precision medicine [59, 60, 61, 62, 63, 64, 65] (Figure 5).

### 1.8 FDA Regulations and Approval of Wearable Medical Devices

The widespread use of digital tools in healthcare today raises a number of practical issues, such as technical specificities, privacy and data security, evaluation of clinical safety and efficiency, patient benefits in terms of rules and legal frameworks, and market access for such tools. The data required for the functionality of many digital tools is collected by sensors. As a result, they are available in a wide range of forms and functions and address the broadest imaginable range of health‐related issues and medical conditions, including cardiovascular health.

Wearable medical devices in the United States are subject to regulatory oversight by the U.S. Food and Drug Administration (FDA), which classifies them based on risk into Class I, II, or III, with corresponding requirements for premarket notification [510(k)], premarket approval (PMA), or exemption [66]. Key regulations include compliance with 21 CFR Part 11 for electronic records and signatures, 21 CFR Part 820 for Quality System Regulation (QSR), and adherence to medical device reporting requirements under 21 CFR Part 803. Internationally, ISO 13485:2016 sets the standard for quality management systems specific to medical devices, ensuring consistent design, development, production, and post‐market surveillance [66]. Additionally, wearable devices must comply with IEC 60601 for safety and performance of medical electrical equipment, and ISO 14971 for risk management, while demonstrating cybersecurity measures as guided by FDA recommendations to protect patient data integrity, confidentiality, and availability throughout the device lifecycle [66].

The field of cardiovascular medicine is increasingly utilizing digitally driven technologies. While the development of medical products is advancing quickly and enabling new uses in cases of cardiac monitoring and other areas, the regulatory and legal requirements that govern market access are frequently evolving slowly and occasionally posing barriers to the market [43, 45, 65, 66, 67, 68, 69, 70, 71]. Their market access through certification or authorization is the most crucial regulatory factor for wearables used for cardiovascular diseases.

### 1.9 Reimbursement Considerations and Insurance Coverage

Who will pay for the purchase and continuous use of a gadget, and in what form, is one of the most crucial considerations for device producers. The response is heavily influenced by the local health care system, which in turn frequently establishes requirements for using the devices in actual practice (beyond what is required by local regulatory bodies).

These FDA‐approved wearable devices are typically covered by major commercial health plans since they are used to identify or treat conditions. Additionally, private insurance frequently pays for the doctor's analysis of the data. Personal cardiac monitoring devices not prescribed by a physician are usually not covered by insurance. This is usually because there is not enough evidence that the device is necessary for a patient's care.

### 1.10 Emerging Trends and Future Outlook of Wearable Technologies

Numerous wearables are available that can track a wide range of health and activity metrics. The devices that are currently in the market are worn on the fingers, wrists, arms, chest, and, in the case of continuous glucose monitors (CGMs), subcutaneously [37, 38, 39, 40, 41, 42]. Even though they were initially designed to track activity, more recent gadgets can also track sleep, temperature, energy usage, multiple cardio‐respiratory parameters, and even dynamic metabolic physiology. This industry, which mostly emerged from Silicon Valley and was connected to a movement known as “the quantified self,” has benefited from the focus on wellness.

Self‐monitoring wearable technologies are being promoted as a tool for serious sports as well as for daily health monitoring and lifestyle enhancement [37, 38]. They have grown in popularity and sophistication. Physicians have just recently begun to realize the potential value of their expertise in cardiovascular care, sometimes as a result of their patients' unexpected disclosure of information. Perhaps it is now inevitable that wearables will become an integral part in today's healthcare [45, 46, 47, 48].

The WHO estimates that more than 17 million people globally die from CVDs each year, which is equal to half of all fatalities in the US [39]. Healthcare systems around the world struggle with the rising costs of medical services and treatments; nevertheless, remote patient monitoring through wearable technology can lower CVD management costs and provide better patient outcomes. In other words, a promising alternative for rapid and accurate medical follow‐up of patients with CVDs or those at high risk of acquiring them is the use of portable and discrete monitoring equipment in conjunction with telecommunication technology [62, 63, 64, 65, 66, 67, 68].

Noninvasive glucose monitoring in wearables is gaining attention because of the strong link between impaired glucose metabolism, diabetes, and cardiovascular disease risk. Innovations such as low‐cost portable microwave sensors utilizing a four‐cell complementary split‐ring resonator (CSRR) hexagonal configuration enable accurate glucose measurement without subcutaneous sensors, reducing discomfort and improving long‐term adherence. For patients with or at risk of cardiovascular disease—particularly those with metabolic syndrome or diabetes—these wearables could facilitate continuous metabolic monitoring alongside heart rate, blood pressure, and vascular health parameters. This integration would allow for more comprehensive cardiovascular risk assessment and early intervention, aligning with the move toward proactive, personalized, and data‐driven cardiac care[72].

## 2 Conclusion

Wearable technology is reshaping cardiovascular care by transforming subjective patient recall into continuous, objective data on heart health, physical activity, and lifestyle habits. Beyond monitoring, these devices can drive behavioral change—encouraging regular exercise, better sleep, and healthier routines that directly reduce cardiovascular risk. While not a substitute for active living, real‐time feedback and goal tracking can help even sedentary individuals adopt heart‐healthy habits. With ongoing advances in sensors and analytics, wearables are poised to become essential tools for proactive prevention and long‐term cardiovascular wellness.

## Author Contributions

**Abubakar Nazir:** Conceptualization, writing – original draft, writing – review and editing, project administration, supervision, validation. **Awais Nazir:** Writing – original draft, writing – review and editing. **Muhammad Shah Wali Jamal:** Writing – original draft, writing – review and editing. **Safi ur Rehman Sadiq:** Writing – original draft, writing – review and editing. **Shafaq Aman:** Writing – original draft, writing – review and editing. **Mubarak Jolayemi Mustapha:** Writing – original draft, writing – review and editing, supervision. **Sodiq Olatunbosun Lawal:** Writing – original draft, writing – review and editing. **Misbahudeen Olohuntoyin AbdulKareem:** Writing – original draft, writing – review and editing. **Mustapha Fatihi Bamigbola:** Writing – original draft, writing – review and editing.

## Ethics Statement

The authors have nothing to report.

## Conflicts of Interest

The authors declare no conflicts of interest.

## Transparency Statement

The lead author Abubakar Nazir affirms that this manuscript is an honest, accurate, and transparent account of the study being reported; that no important aspects of the study have been omitted; and that any discrepancies from the study as planned (and, if relevant, registered) have been explained.

## Data Availability

Data sharing not applicable to this article as no datasets were generated or analyzed during the current study.
